# A Study on the Electromagnetic–Thermal Coupling Effect of Cross-Slot Frequency Selective Surface

**DOI:** 10.3390/ma15020640

**Published:** 2022-01-15

**Authors:** Yi Lu, Juan Chen, Jianxing Li, Wenjing Xu

**Affiliations:** 1 School of Electronic and Information Engineering, Xi’an Jiaotong University, Xi’an 710049, China; ly8023@stu.xjtu.edu.cn (Y.L.); jianxingli.china@xjtu.edu.cn (J.L.); xwj5408@stu.xjtu.edu.cn (W.X.); 2 Shenzhen Research School, Xi’an Jiaotong University, Shenzhen 518000, China

**Keywords:** electromagnetic–thermal coupling effect, frequency selective surface (FSS), high-power microwave (HPM) applications, multi-field coupling, slot structure

## Abstract

In high-power microwave applications, the electromagnetic-thermal effect of frequency selective surface (FSS) cannot be ignored. In this paper, the electromagnetic-thermal coupling effects of cross-slot FSS were studied. We used an equivalent circuit method and CST software to analyze the electromagnetic characteristics of cross-slot FSS. Then, we used multi-field simulation software COMSOL Multiphysics to study the thermal effect of the FSSs. To verify the simulation results, we used a horn antenna with a power of 20 W to radiate the FSSs and obtain the stable temperature distribution of the FSSs. By using simulations and experiments, it is found that the maximum temperature of the cross-slot FSS appears in the middle of the cross slot. It is also found that the FSS with a narrow slot has severer thermal effect than that with a wide slot. In addition, the effects of different incident angles on the temperature variation of FSS under TE and TM polarization were also studied. It is found that in TE polarization, with the increase in incident angle, the maximum stable temperature of FSS increases gradually. In TM polarization, with the increase in incident angle, the maximum stable temperature of FSS decreases gradually.

## 1. Introduction

Multi-physical field coupling problem means that in practical engineering applications, there are many physical fields at the same time, such as electromagnetic (EM) field, thermal field, stress field, humidity field, etc., which are always superimposed and affected by each other. Although some of these physical fields are dominant, and some of them are secondary (usually ignored by researchers), for some specific engineering applications, detailed analysis of the multi-field coupling is of great significance for accurate design and high-quality control. In the EM field, EM-thermal coupling is the most common form of multi-physical field coupling. In [1], the coupled structural–electromagnetic-thermal (SET) model of active phased array antenna (APAA) is established to study the influence of different temperatures and different structure distortions on the EM performance of APAA. In [2], an electromagnetic–thermal-stress co-simulation method is proposed to predict the radiation characteristics and other performance of a patch antenna. The results show that the radiation efficiency of the patch antenna decreases significantly due to the temperature rise and deformation. In [3], a supercomputing-based parallel domain decomposition method is used to simulate the EM-thermal co-simulation of a large antenna array. It can be seen that most of the current research about the EM-thermal effect focuses on the analysis of the performance of various kinds of antennas. However, in some high-power microwave (HPM) applications, the EM-thermal effect of frequency selective surface (FSS) cannot be ignored either.

FSS is a periodic planar array structure. A typical FSS consists of some metal patches or slots arranged in a certain way, which is essentially equivalent to a spatial filter. FSSs show obvious band-pass [4,5,6] or band-stop [7,8,9,10] EM transmission performance. The reasonable design of unit pattern shape, unit gap, arrangement, and dielectric loading can make FSSs have good frequency selectivity and polarization stability. FSSs can effectively control the transmission and reflection of the EM wave.

FSS is widely used in radomes, filter radomes, EM absorbers, and other engineering and military applications [11]. In the traditional design process, researchers usually consider whether the EM characteristics of FSS meet the application requirements, ignoring the influence of other physical fields on its characteristics. However, when a high-power EM wave or strong EM pulse radiates on the surface of FSS, the EM energy loss often causes the temperature rise in the FSS structure, which will bring the mutual coupling of EM field and thermal field. When the temperature is very high, it will affect the stability of the communication system and, meanwhile, lead to the breakdown of the FSS structure [12]. For example, FSS with band-pass characteristics can be placed in the front end of the radar system or integrated into the radome of a base station antenna to select specific bands of EM waves to transmit or receive. However, when natural lightning occurs or when artificial HPM weapons are used in the military, the FSS structure may be damaged [13,14,15]. Therefore, the analysis of the EM–thermal coupling effect of FSS under HPM incidence is of great significance in practical engineering applications.

There are two difficulties in studying the EM-thermal effect of FSS. Firstly, in order to use the numerical software to simulate the EM-thermal effect accurately, we need the variation of the materials parameters (such as dielectric constant) with the temperature and frequency. However, in most cases, this property is difficult to analyze and measure in the radio frequency band, which results in difficulty in the numerical simulation for the EM-thermal effect. In [16], a statistical approach called the stochastic collocation method (SCM) is used to solve the uncertainty of input parameters in computational electromagnetics. This method is worthy of reference, but it is still unable to accurately simulate the variation of specific material parameters with frequency and temperature. Secondly, the simulation conditions of EM–thermal coupling are difficult to match with the experimental environment. For example, there is often a loss of energy in an experiment, and it is difficult to determine the actual boundary conditions of heat transmission in the experiment. Therefore, there is often a gap between the software simulation results and the actual experimental results. Due to the difficulties mentioned above, there are very few studies about the thermal effect of FSSs or metamaterials in HPM systems. 

In [17], M. Li et al. defined a factor called maximum field enhancement factor (MFEF) to evaluate the power handling capability of FSSs in HPM applications. In [18], C. Liu et al. studied the influence of microwave breakdown on the frequency response of metamaterials in HPM application. In [19], Seviour et al. studied the thermal effect of split-ring resonators (SRRs) exposed to relatively high-power EM waves. The results show that the structure will burn when exposed to 1 W EM waves, which means that the traditional metamaterials are not suitable for HPM applications. In [20], In et al. studied the effect of joule heat on the absorptivity of metamaterial absorber and proposed that temperature change may affect the dielectric constant of materials, thus reducing the absorptivity of metamaterial absorber. In [21], the authors demonstrated that when the FSS is irradiated at high power, a breakdown at one location may cause a breakdown at another location, where the local electric field intensities are not high enough to cause breakdown under normal circumstances. In fact, these studies only focus on the EM characteristics of the metamaterials and verify the thermal effect or power tolerance by experiments. They did not conduct a coupling analysis on the EM-thermal effect.

In this paper, we used the multi-field simulation software COMSOL Multiphysics to study the EM-thermal coupling effect of the cross-slot FSS structure. Cross-slot structure is one of the most common structures in FSS design. The FSSs of Jerusalem cross-slot structure [22,23,24], Swastika structure [25,26], and Maltese cross-slot structure [27] can be regarded as the deformation of the cross-slot structure. Therefore, in order to make the study of the EM-thermal effect of FSS universal and referential, we study typical cross-slot FSS structures. 

In Section 2, the relevant algorithm of EMthermal coupling, the design of cross-slot FSS, and EM characteristics and the thermal effect of the designed FSS are analyzed. It is concluded that the FSS with a narrow slot has severer thermal effect than that with a wide slot. In Section 3, the EM–thermal effect of the FSS is verified experimentally. The experimental results show that EM characteristics and the thermal effect of FSS are consistent with simulation results. Final conclusions are presented in Section 4.

## 2. Research Methods and FSS Design

### 2.1. Electromagnetic-Thermal Coupling Algorithm

The EM-induced heating pattern in a medium under EM radiation can be obtained from the heat transfer equation (HTE) [28]. The equation is

(1) $$\rho C_{P}\frac{\partial T(x,y,z,t)}{\partial t}=k_{t}\nabla^{2}T(x,y,z,t)+P_{d}(x,y,z,t)$$

where *ρ*—medium density (kg/m^3^); *C_p_*—specific heat of the medium (J/(kg∙K)); *T*(*x*,*y*,*z*,*t*)—medium temperature (K); *k_t_*—thermal conductivity of the medium (W/(m∙K)); *P_d_*(*x*,*y*,*z*,*t*)—electromagnetic power dissipated per unit volume (W/m^3^).

In addition, the convective boundary condition can be used at the surfaces of the heated materials. The condition can be written as

(2) $$\frac{\partial T}{\partial n}=\frac{h}{k_{t}}(T_{ext}-T_{surf})$$

where *h*—Convection heat transfer parameter (W∙m^−2^∙K^−1^); *n*—unit vector normal to the surface at the considered point; *T_surf_*—surface temperature of the material (K); *T_ext_*—temperature of the surrounding external medium (K).

From Equation (1), it can be seen that the key to EM-thermal coupling is the EM dissipated power, $P_{d}$. $P_{d}$ consists of two parts, one is the resistance loss, $P_{e}$. The other part is magnetic loss, $P_{h}$. The calculation formulas are as follows:

(3) Pe=12Re(J⋅E*)=12σ|E|2

(4) Ph=12Re(iωB⋅H*)

(5) $$P_{d}=P_{e}+P_{h}$$

where *E*—electric field intensity (V/m); *J*—conduction current density (A/m^2^); *σ*—conductivity (S/m); *H*—magnetic field intensity (A/m); *B*—magnetic induction (T); *i*—imaginary unit; *ω*—angular frequency (rad/s).

In general, there is no magnetic dispersion in the permeability at microwaves and higher frequencies, so the magnetic loss $P_{h}$ can be ignored. The dissipated power $P_{d}$ is mainly determined by $P_{e}$. Taking Equation (3) into Equation (1), we can obtain

(6) $$\rho C_{P}\frac{\partial T(x,y,z,t)}{\partial t}=k_{t}\nabla^{2}T(x,y,z,t)+\frac{1}{2}\sigma {\left|E\right|}^{2}$$

For lossy medium, we generally consider its loss tangent rather than conductivity. According to the conversion relationship between loss tangent and conductivity, Equation (6) becomes

(7) $$\rho C_{P}\frac{\partial T(x,y,z,t)}{\partial t}=k_{t}\nabla^{2}T(x,y,z,t)+\frac{1}{2}\omega \varepsilon_{0}\varepsilon_{r}\tan\delta {\left|E\right|}^{2}$$

where $\varepsilon_{0}$—vacuum dielectric constant (F/m); $\varepsilon_{r}$—the relative permittivity of the medium; tanδ—Loss tangent of the medium.

From the above analysis, it can be seen that when solving the EM-thermal coupling problem, Maxwell equations are used to calculate the electromagnetic field firstly, and then, $P_{d}$ is calculated by using Equations (3)–(5). By taking $P_{d}$ to Equation (1) and combining the boundary condition of Equation (2), the thermal field can be calculated. The relationship between electric field and thermal field is directly illustrated in Equation (7).

### 2.2. Structure of FSS

**Figure 1:**
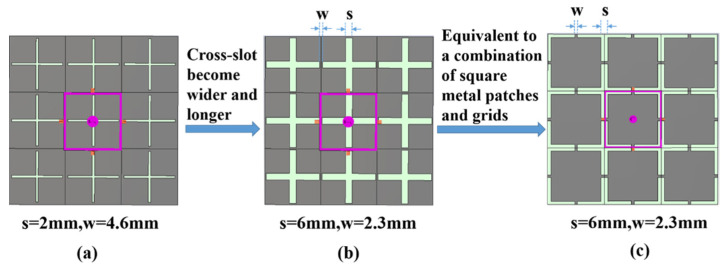
3 × 3 units cross-slot FSS structures: (**a**) cross-slot FSS with slot width of 2 mm; (**b**) cross-slot FSS with slot width of 6 mm; (**c**) a combination of square metal patches and grids, it is equivalent to (**b**).

Figure 1 shows typical cross-slot FSS structures. The gray part represents the metal, and the green part represents the dielectric. The FSS unit is a square with a side length of 50 mm. Figure 1a,b show two FSS structures with different slot sizes. The cross-slot structure is equivalent to a combination of square metal patches and grids (the short thin wires), as shown in Figure 1c, where w is the width of the metal grid, and s is the distance between the square metal patch units and is also the width of the slot. The cross-slot FSS shows band-pass characteristics because the patches and grids can be equivalent to capacitance and inductance, respectively (the detailed analysis is in Section 2.3). The cross-slot structure is printed on an FR-4 dielectric substrate whose thickness is 1 mm, the dielectric constant is 4.3, and the loss tangent is 0.025.

### 2.3. Analysis of EM Effect of FSS

**Figure 2:**
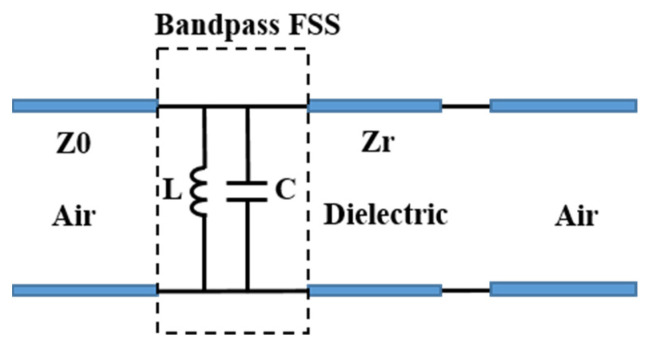
Equivalent circuit diagram of the cross-slot FSS.

Cross-slot FSS shows band-pass characteristics, which can be qualitatively analyzed by an equivalent circuit method. It is well known that a parallel LC circuit acts as a band-pass filter [29,30,31,32]. According to references [17] and [33], the dielectric substrate can be equivalent to a section of a transmission line. A periodic square metal patch can be equivalent to a parallel capacitance, and the periodic metal grid can be equivalent to a parallel inductance. Therefore, the equivalent circuit of the cross-slot FSS is an LC parallel circuit [32]. Figure 2 is the equivalent circuit diagram of the cross-slot FSS. Z0 and Zr represent the free space wave impedance, and the dielectric equivalent transmission line impedance, respectively. Its resonant frequency *f_0_* and relative bandwidth Δ*f/f_0_* are expressed by the following two formulas:

(8) $$f_{0}=\frac{1}{2\pi \sqrt{LC}}$$

(9) $$\frac{\Delta f}{f_{0}}\propto \sqrt{\frac{L}{C}}$$

Although it is difficult to accurately calculate the magnitude of L and C, this approximation provides simple yet qualitative insight into the filter’s characteristics. From reference [17], Equations (8) and (9), it can be seen that when w is smaller, the equivalent inductance L will be larger. Thus, the resonant frequency *f_0_* will be smaller, and the bandwidth *∆f* will be wider. On the other hand, when s is larger, the equivalent capacitance C will be smaller, which results in *f_0_* becoming larger, and *∆f* will be wider.

**Figure 3:**
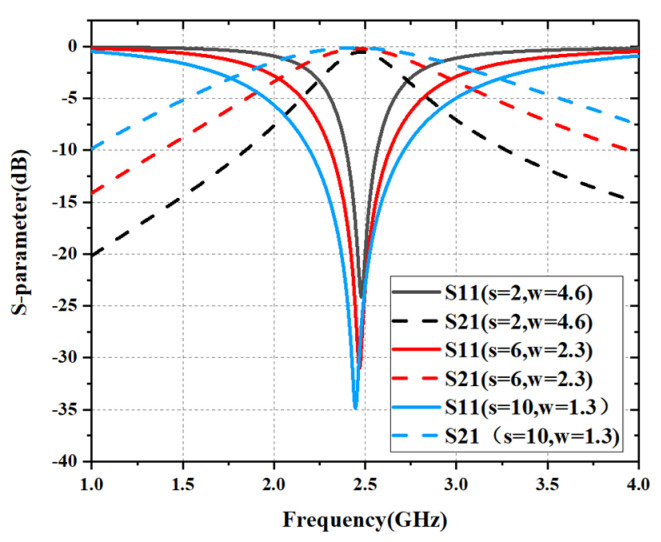
S-parameters of FSSs with different values of s and w (mm).

We used the EM simulation software CST to study the EM characteristics of the cross-slot FSS, and study the S parameters with different s and w parameters. The simulation results are shown in Figure 3. As the FSS structure is completely symmetric, the S parameters of TE and TM polarization are the same in the case of normal incidence. As can be seen from Figure 3, the FSSs resonate at 2.45 GHz. When s increases and w decreases—namely, the slot becomes wider and longer, resonant frequency *f_0_* is unchanged, but the bandwidth *∆f* increases obviously. This is consistent with the results of the equivalent circuit analysis shown in Figure 2.

### 2.4. Analysis of Thermal Effect of FSS

The EM-thermal effect of FSS was mainly analyzed by means of the multi-field simulation software COMSOL Multiphysics 5.4 from COMSOL Group in Stockholm, Sweden. EM-thermal coupling is actually a two-way coupling process. EM field causes the change of temperature. When the temperature changes, some material properties (such as dielectric constant and conductivity) change accordingly, which will bring some effect on the EM field. In this paper, because the properties of FR-4 are not sensitive to temperature, we only considered the unidirectional coupling from the EM field to the thermal field and ignored the influence of the thermal field on the EM field. However, it cannot be ignored that the glass transition temperature (Tg) of the commonly used FR-4 material is about 140 °C. When the temperature is above 140 °C, its mechanical strength will be significantly worse, and it can no longer be used normally. Therefore, our main purpose was to find the maximum radiation power that FSS can withstand when its temperature reaches Tg.

**Figure 4:**
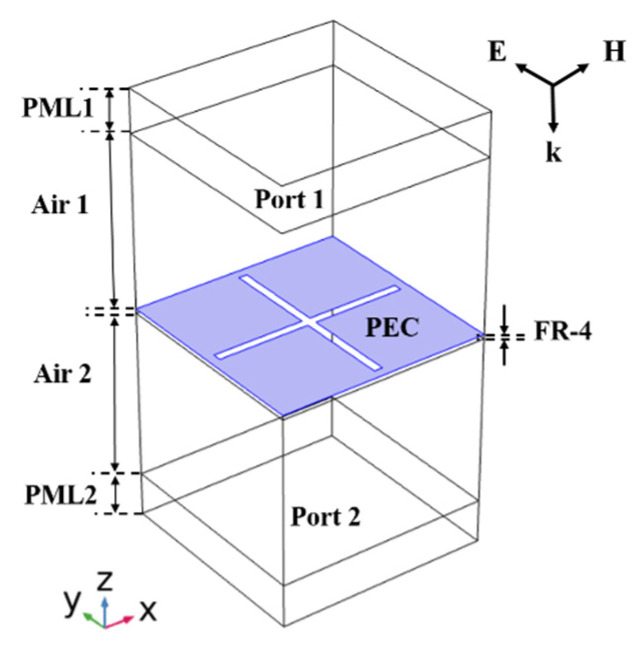
The cross-slot FSS structure modeled in COMSOL.

Figure 4 shows the cross-slot FSS structure modeled in COMSOL. From the top to the bottom, the model consists of the perfectly matched layer (PML1), air (Air 1), FSS structure, air (Air 2), and perfectly matched layer (PML2). Firstly, the boundary conditions of the model were set in the “electromagnetic waves, frequency domain” module. Floquet–periodic boundary conditions were used on four sides of the unit cell to simulate the infinite 2D array. Perfectly matched layers (PMLs) on the top and bottom of the unit cell absorbed the excited mode from the source port and any higher-order modes generated by the periodic structure. The thickness of the PMLs is generally set to about 0.5−1 λ, which was set as 10 mm in this paper. The upper boundary condition of PML1 is the PEC boundary, and the lower boundary condition of PML2 is the scattering boundary condition. The upper boundary of Air 1 is input port 1. The lower boundary of Air 2 is output port 2. As the simulated structure was periodic, both ports were set to periodic ports. The thickness of the two air layers was set as 40 mm. The middle FSS structure was a single-layer, cross-slot structure on the FR-4 substrate, and the upper surface (purple area) was PEC. The FR-4 material has a thickness of 1 mm. Its dielectric constant is 4.3, the loss tangent is 0.025, the specific heat capacity is 600 J/(kg·K), the density is 1800 kg/m^3^, and the thermal conductivity is 0.3 W/(m·K). 

Next, the heat transfer boundary conditions of the model were set in the “heat transfer in solids” module. As we only focused on the thermal field of FSS, we only put the FSS structure into this module. The boundary conditions around the FSS were set as periodic conditions, and the upper and lower boundary conditions of the FSS were convective heat flux boundary conditions. The convective heat flux boundary condition considers the convective heat exchange between the heating material and the surrounding environment. It is determined by setting appropriate values of the convection heat transfer parameter h and the external environment temperature text in Equation (2). When the air is naturally convective, h is generally 5–25 W·m^−2^·K^−1^. In the simulation, we take h = 10 W·m^−2^·K^−1^ and Text = 20 °C.

In this study, we used a frequency-domain solver to calculate the EM field at 2.45 GHz and used the transient solver to calculate the thermal field within 0–1000 s. The mesh grid was controlled by the physical field preset by COMSOL. The maximum grid cell size was 1/5 λ.

**Figure 5:**
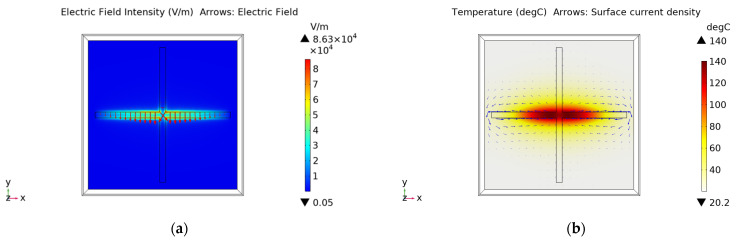
The distribution of (**a**) electric field and (**b**) stable thermal field of the FSS with 2 mm slot.

Figure 5 shows the distribution of the electric field and stable thermal field of the FSS with the slot width of 2 mm under 5.5 W (0.22 W/cm^2^) power radiation. It can be seen that the electric intensity is concentrated in the middle of the slot. Compared with the distribution of electric field in [12,18], we can find that the maximum electric field of the slot structure is indeed distributed in the middle of the slot. The maximum temperature appears in the center of the cross slot, rising from the initial temperature of 20 °C to the stable temperature of about 140 °C. Based on Equation (7), it can be found that the dissipated power density is the largest where the electric field intensity distribution is strongest, so the temperature rise in this part is the most obvious.

**Figure 6:**
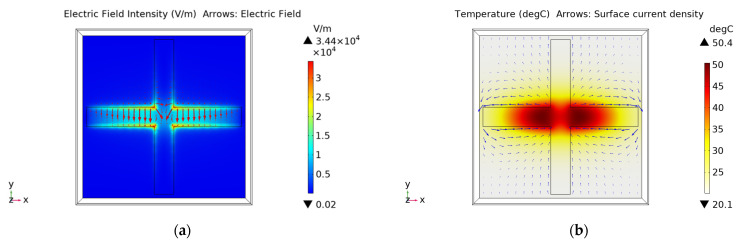
The distribution of (**a**) electric field and (**b**) stable thermal field of the FSS with 6 mm slot.

Figure 6 shows the distribution of electric field and stable thermal field of FSS with 6 mm slot width under the same 5.5 W (0.22 W/cm^2^) power radiation. Compared with Figure 5, it can be found that the wider and longer the cross slot is, the lower the electric field intensity and temperature are. When the slot width is 6 mm, the maximum stable temperature is only 50.4 °C; that is to say, under the same power radiation, the maximum temperature of the FSS with a 2 mm slot has reached Tg and can no longer be used, while the maximum temperature of the FSS with a 6 mm slot is far lower than Tg and can continue to be used. In fact, through simulations, it can be found that when the incident power is 22 W (0.88 W/cm^2^), the maximum temperature of the FSS with a 6mm slot width can reach Tg. Table 1 shows the maximum power that three groups of FSS with different values of s and w parameters can withstand when reaching 140 °C. This indicates that the wider and longer the slot is, the greater the power handling capability of the FSS is. This phenomenon can be qualitatively analyzed by using the equivalent circuit shown in Figure 2. When the cross slot is wider and longer, the equivalent inductance L will be larger, and the equivalent capacitance C will be smaller. Therefore, the total reactance of the parallel LC circuit increases, and the current through the circuit decreases correspondingly. It is equivalent to the temperature reduction in the FSS structure. This can also be explained more intuitively from the perspective of dissipated power density. The narrower the slot is, the greater the electric field intensity in the slot is. This results in an increase in the dissipated power density, which makes the thermal effect more obvious and the power handling capability worse.

**Figure 7:**
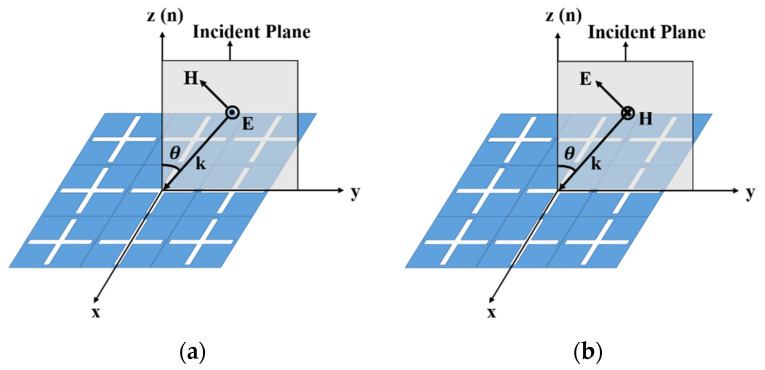
The concept of TE/TM polarization: (**a**)TE polarization;(**b**) TM polarization.

In addition to studying the case of normal incidence, we also studied the influence of oblique incidence on the temperature rise of FSS in TE/TM polarization. Figure 7 shows the concept of TE/TM polarization. When the EM wave is obliquely incident on a dielectric plane, the wave vector direction (**k**) and the normal direction (**n**) of the plane form a plane (yz plane), which is called the incident plane. If the electric field direction is perpendicular to the incident plane, we denote it as oblique incidence under TE polarization, as shown in Figure 7a. If the electric field direction is parallel to the incident plane, we denote it as oblique incidence under TM polarization, as shown in Figure 7b. Taking Figure 7 as an example, for TE polarization, the direction of the electric field remains unchanged, and the direction of the magnetic field changes from −y direction to +z direction with the increase in incident angle θ. For TM polarization, the direction of the magnetic field remains unchanged, and the direction of the electric field changes from −y direction to +z direction with the increase in incident angle θ.

**Figure 8:**
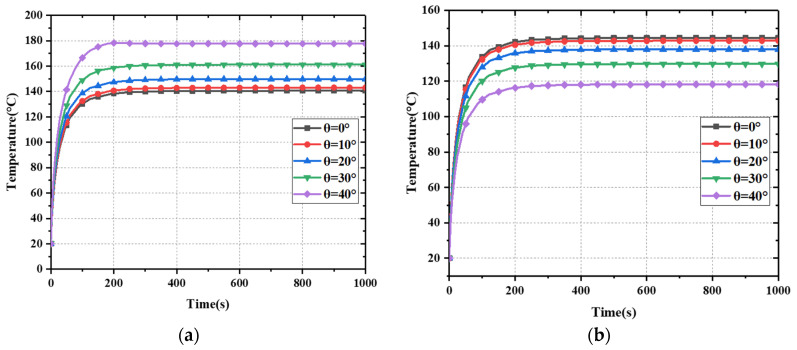
Time dependence of maximum temperature at different incident angles under (**a**) TE polarization and (**b**) TM polarization.

Figure 8 shows the maximum temperature of the FSS with different incident angles under TE and TM polarization. Here, the width of the slot is 2 mm, and the incident power is 5.5 W (0.22 W/cm^2^). It can be seen that in TE polarization, with the increase in incident angle θ, the maximum stable temperature of FSS increases gradually. In TM polarization, with the increase in incident angle θ, the maximum stable temperature of FSS decreases gradually. This is because, in TE polarization, the electric field intensity of the FSS surface increases with the increase in incident angle, while in TM polarization, the electric field intensity decreases with the increase in the incident angle. 

## 3. Experiment Demonstration

### 3.1. FSSs Processing and the Simulation of Experimental Environment

**Figure 9:**
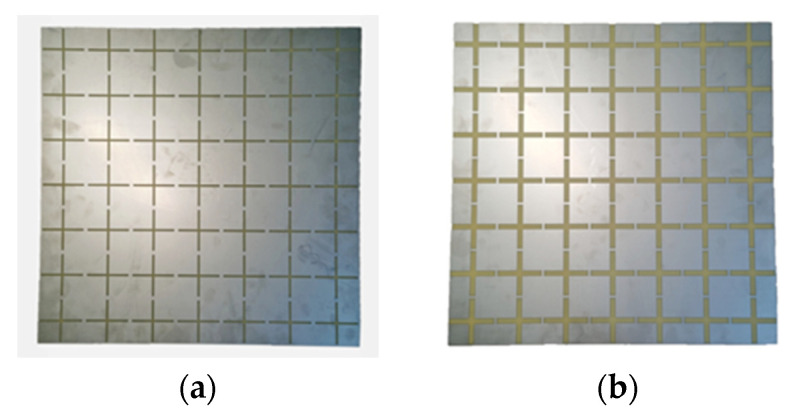
The fabricated 7 × 7 units FSSs samples: (**a**) s = 2 mm, w = 4.6 mm; (**b**) s = 6 mm, w = 2.3 mm.

The FSSs were fabricated and tested experimentally. The fabricated samples of FSSs with a slot width of 2 mm and 6 mm are shown in Figure 9.

**Figure 10:**
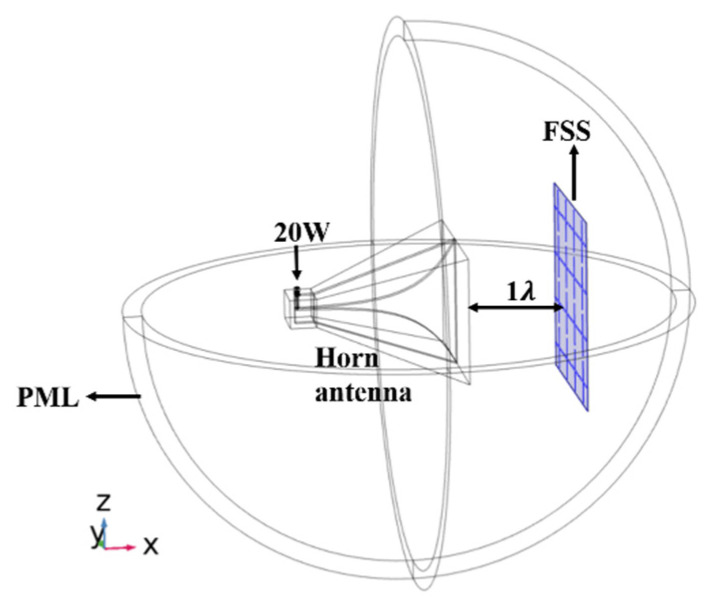
The overall model of horn antenna and FSS.

**Figure 11:**
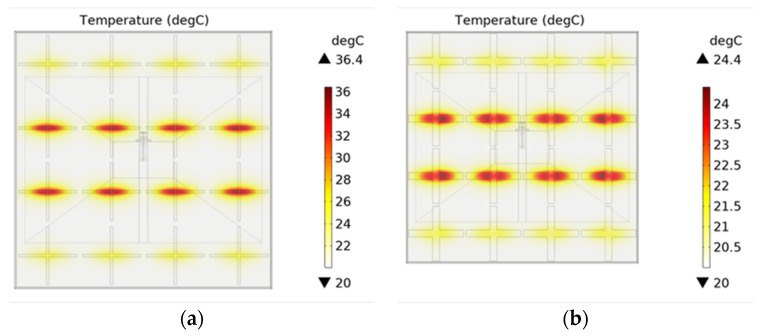
The stable thermal field distribution: (**a** ) = 2 mm, w = 4.6 mm; (**b**) s = 6 mm, w = 2.3 mm.

Since it is impossible to make an infinite periodic FSS structure in practice, we needed to simulate the actual experimental environment numerically. A horn antenna radiated the FSS structure, as shown in Figure 10. The input power of the horn antenna was set to 20 W due to the limitation of experimental equipment. The distance between the horn surface and the FSS surface is 12 cm (about 1 λ). The horn antenna and the FSS were surrounded by a spherical PML to simulate the infinite free space. Figure 11a,b show the stable thermal field distribution after 1000 s, with the slot width of 2 mm and 6 mm, respectively. We can compare the results in Figure 11 with those in Figure 5b and Figure 6b. Figure 5b and Figure 6b simulate the thermal distribution of infinite periodic FSS structure under uniform plane wave radiation. The thermal distribution should be consistent for each cell structure because periodic boundaries are used. Figure 11 simulates the thermal distribution of the EM wave radiated by the horn antenna on the finite size FSS sample. It can be seen that due to the directivity of the horn antenna, the temperature of the FSS unit located in the radiation center of the horn antenna is higher than that of the edge FSS unit. However, in both cases, the highest temperature occurs in the middle of the cross slot. Moreover, it is obvious that the stable temperature of the FSS with a slot width of 2 mm is higher than that of the FSS with a slot width of 6 mm. This indicates that no matter whether the incident EM wave is evenly distributed or not, the middle of the slot is the part that is most prone to damage or breakdown for cross-slot FSS.

### 3.2. The S Parameters Test

**Figure 12:**
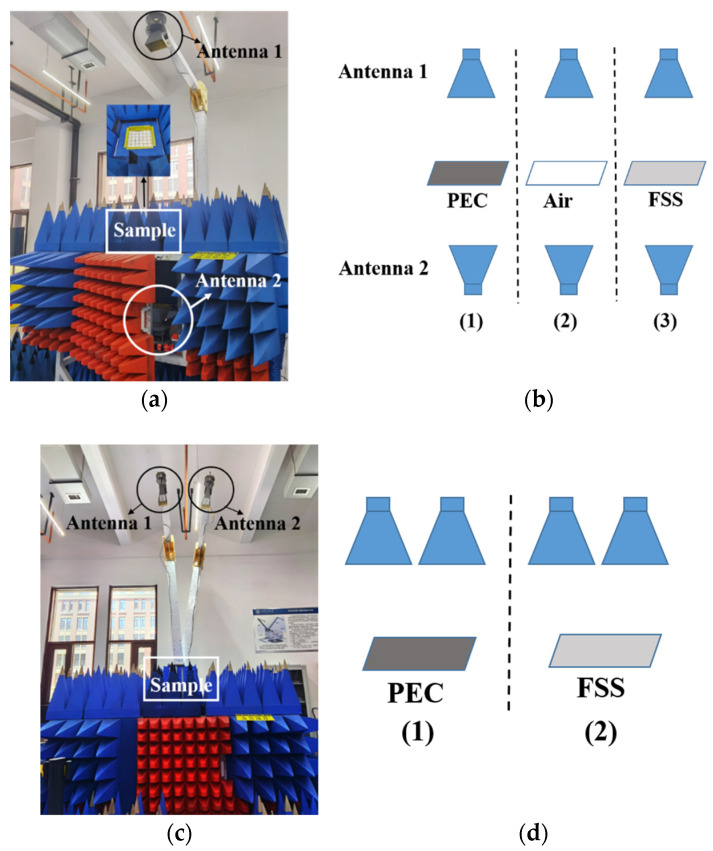
Test scene and test process of S parameters:(**a**) test scene of S21; (**b**) test process of S21; (**c**) test scene of S11; (**d**) test process of S11.

The test scene and test process of S parameters are shown in Figure 12. In Figure 12a, two standard gain horn antennas were placed above and below the FSS, respectively, to test the S21. The two antennas were connected to a vector network analyzer. To minimize the impact of the surrounding environment on the test results, the test process was divided into three steps, as shown in Figure 12b. First, we put a metal plate (PEC) with the same size as the measured FSS structure between the two antennas. At this time, the test was completely without transmission. In the second step, we took away the metal plate. At this time, we tested the complete transmission. In the third step, we put the FSS sample between the two antennas. At this time, we tested the transmission of the FSS. By normalizing the results of the first and second steps to “0” and “1”, respectively, and then processing the results of the third step in proportion, we obtained the transmission results (S21). Similarly, when testing the reflection, we put the two standard gain horn antennas on the same side, as shown in Figure 12c. The test process was divided into two steps, as shown in Figure 12d. First, we tested the reflection of the metal plate. At this time, the test result should be total reflection. Then, we tested the reflection of the FSS sample. Through the normalization post-processing of the two sets of data, the test result of PEC was treated as a total reflection “1”, and the test result of FSS was treated in the same proportion to obtain its reflection parameter S11.

**Figure 13:**
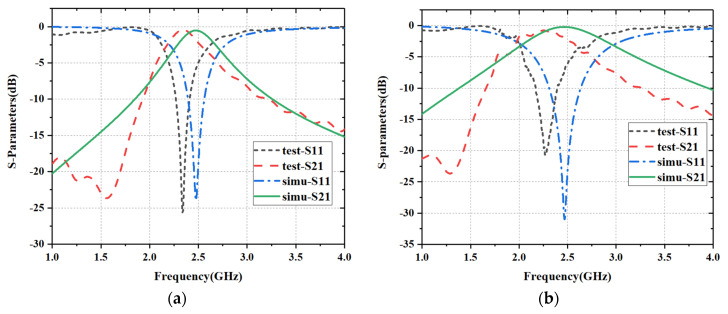
Comparison of S-parameters: (**a**) s = 2 mm, w = 4.6 mm; (**b**) s = 6 mm, w = 2.3 mm.

Figure 13 shows the comparison of the S parameters between test results and simulation results. Due to the machining error and test error, the frequency selection point of FSSs has a little deviation, but it is still within a reasonable range because the test results of these two samples both meet the band-pass characteristics at 2.45 GHz.

### 3.3. Temperature Test

**Figure 14:**
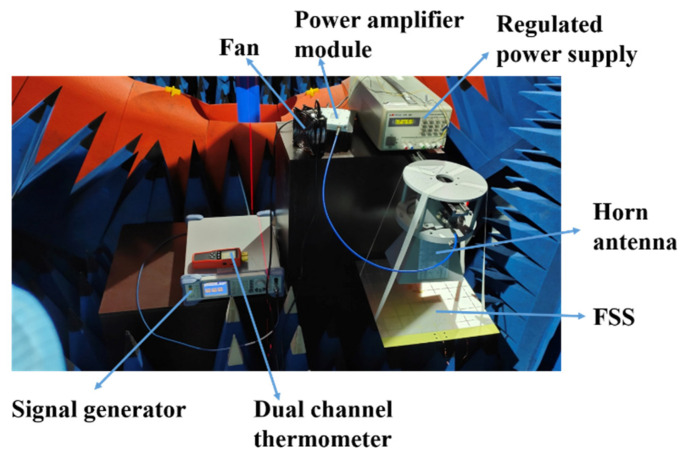
The temperature test scene of FSS.

**Figure 15:**
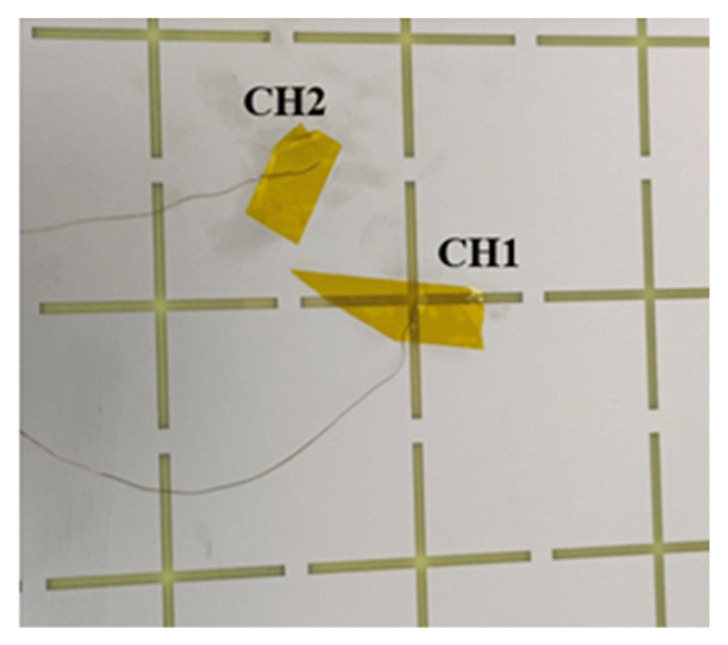
Bonding position of thermocouple wire.

The temperature test scene of FSS is shown in Figure 14. A signal generator output 2.45 GHz, 5.3 dBm sinusoidal signal, which was connected to the input port of a power amplifier module. The power amplifier module was powered by a 28 V regulated power supply and amplified the input 5.3 dBm power to 20 W. The output end of the power amplifier module was connected to a horn antenna. The antenna is a 1.72−2.61 GHz standard gain horn antenna, and its gain is about 13 dB at 2.45 GHz. The excitation of the horn antenna is TE10 mode. The FSS structure was suspended under the horn antenna by tape, and the FSS surface was 12 cm away from the horn antenna. Two sections of K-type thermocouple wires were pasted with heat-resistant tape at the center of the cross slot and the center of the metal patch, as shown in Figure 15. The thermocouple wire was connected to a dual-channel thermometer to measure the temperature of the two parts independently (CH1: cross-slot center; CH2: metal patch center). The whole experimental devices are placed in a microwave anechoic chamber.

**Figure 16:**
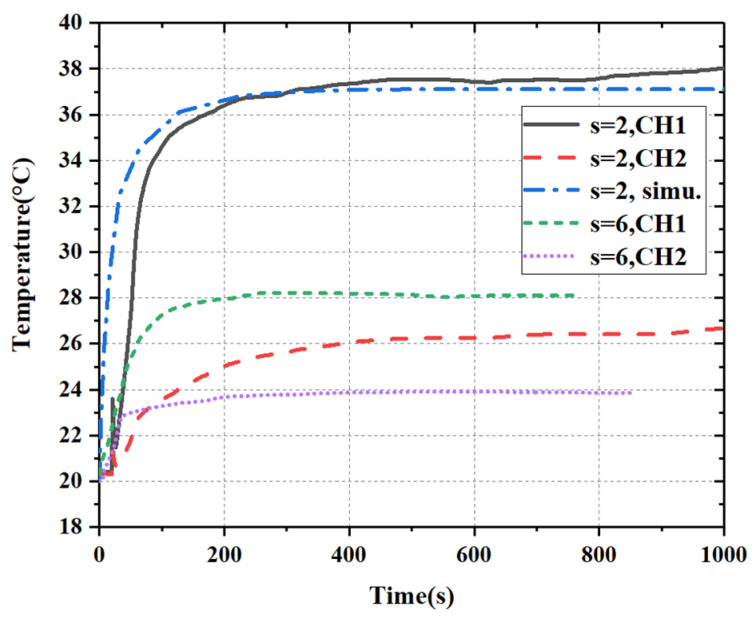
Temperature test results measured by thermocouple.

Since the angle of oblique incidence is hard to be accurately controlled during the test, we only tested the results under the condition of normal incidence (θ=0°). The temperature test results of FSS are shown in Figure 16. It can be seen that the temperature of CH1 is significantly higher than that of CH2 for both kinds of FSSs. In addition, the temperature of the FSS with a 2 mm slot is obviously higher than that with a 6 mm slot, both for CH1 and CH2. These are consistent with the simulation results shown in Figure 11. Additionally, it can be seen that the stable maximum temperature at the cross-slot center reaches 38 °C after about 500 s, which is in good agreement with the simulation result.

**Figure 17:**
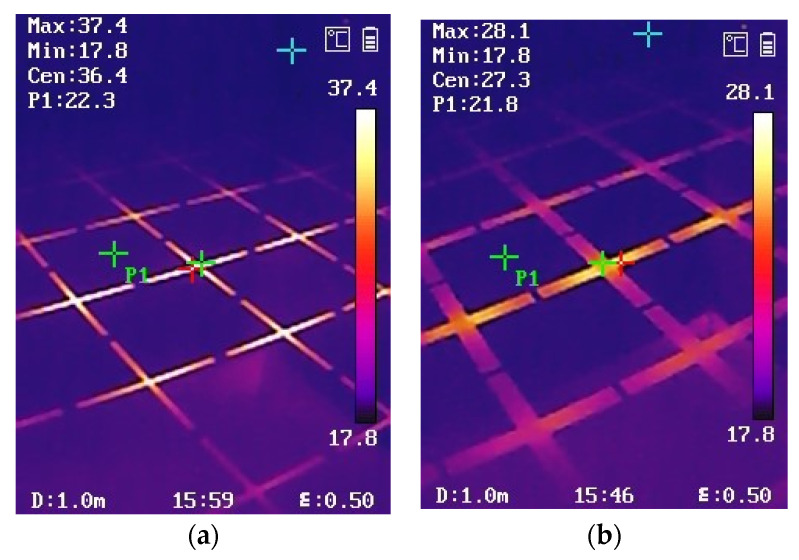
Temperature test results measured by an infrared camera: (**a**) s = 2 mm, w = 4.6 mm; (**b**) s = 6 mm, w = 2.3 mm.

In order to increase the reliability of the experiment, in the same test scene in Figure 14, we also used an infrared camera to test the stable temperature of two kinds of FSS with different slot widths, as shown in Figure 17. Figure 17a shows that, for the FSS with a 2 mm slot, the highest temperature appears at the center of the cross slot, which is 37.4 °C, while in the center of the metal patch (point P1), the temperature is only 22.3 °C. Figure 17 b shows that, for the FSS with a 6 mm slot, the highest temperature appears at the center of the cross slot, which is 28.1 °C, while in the center of the metal patch (point P1), it is only 21.8 °C. The temperature test results in Figure 17 are very consistent with the simulation results in Figure 11, which further verifies the correctness of the simulation.

## 4. Conclusions

The EM–thermal effects of the cross-slot FSS were studied using simulations and verified with experiments. It is found that the maximum temperature of cross-slot FSS is concentrated in the middle of the slot, and the narrower the slot is, the more obvious the temperature effect is. In TE polarization, with the increase in incident angle, the maximum stable temperature of FSS increases gradually. The result is the opposite of that for TM polarization. It is worth mentioning that although the experiments do not provide HPM radiation due to the limitation of experimental equipment, the conclusions obtained from the simulations and experiments are applicable in the HPM field. It shows that to avoid the damage brought by high temperature, the structure with a wider slot width should be selected as far as possible in the design of FSSs working at the same frequency. These conclusions can also be generalized for FSSs with other slot structures. Therefore, this study is significant, as it serves as a guide for the design of slot type FSSs in the HPM field.

## Figures and Tables

**Table 1:** The maximum power at 140 °C of three groups of FSS with different values of s and w (mm).

Structures	S = 2, w = 4.6	S = 6, w = 2.3	S = 10, w = 1.3
Maximum power at 140 °C	5.5 W (0.22 W/cm^2^)	22 W (0.88 W/cm^2^)	51 W (2.04 W/cm^2^)

## Data Availability

The study did not report any data.
